# Viral Appropriation: Laying Claim to Host Nuclear Transport Machinery

**DOI:** 10.3390/cells8060559

**Published:** 2019-06-08

**Authors:** Tanner M. Tessier, Mackenzie J. Dodge, Martin A. Prusinkiewicz, Joe S. Mymryk

**Affiliations:** 1 Department of Microbiology and Immunology, The University of Western Ontario, London, ON N6A 3K7, Canada; ttessie2@uwo.ca (T.M.T.); mdodge@uwo.ca (M.J.D.); mprusink@uwo.ca (M.A.P.); 2 Department of Otolaryngology, Head & Neck Surgery, The University of Western Ontario, London, ON N6A 3K7, Canada; 3 Department of Oncology, The University of Western Ontario, London, ON N6A 3K7, Canada; 4 London Regional Cancer Program, Lawson Health Research Institute, London, ON N6A 5W9, Canada

**Keywords:** virus, nuclear transport, protein localization, infection, antiviral agents, viral mimicry

## Abstract

Protein nuclear transport is an integral process to many cellular pathways and often plays a critical role during viral infection. To overcome the barrier presented by the nuclear membrane and gain access to the nucleus, virally encoded proteins have evolved ways to appropriate components of the nuclear transport machinery. By binding karyopherins, or the nuclear pore complex, viral proteins influence their own transport as well as the transport of key cellular regulatory proteins. This review covers how viral proteins can interact with different components of the nuclear import machinery and how this influences viral replicative cycles. We also highlight the effects that viral perturbation of nuclear transport has on the infected host and how we can exploit viruses as tools to study novel mechanisms of protein nuclear import. Finally, we discuss the possibility that drugs targeting these transport pathways could be repurposed for treating viral infections.

## 1. The Separation of Cytoplasmic and Nuclear Compartments

Within eukaryotic cells the nuclear envelope (NE) provides a physical barrier that spatially separates the contents of the nucleus and cytoplasm at all stages of the cell cycle except during mitosis. The NE confines key cellular processes like transcription and translation to the nuclear and cytoplasmic compartments, respectively. This separation allows for complex regulation of gene expression as well as a variety of other distinguishing factors between prokaryotic and eukaryotic organisms [1,2]. Newly transcribed mRNA must be exported from the nucleus to the cytoplasm to be translated, while nuclear proteins such as transcription factors and histones must be imported into the nucleus from the cytoplasm. For complex biological processes like signal transduction to occur, there must be dynamic spatial and temporal regulation of proteins and other macromolecules between the cytoplasmic and nuclear compartments, which ultimately requires passage across the NE in one or more directions. 

Movement of molecules across the NE is restricted to the nuclear pore complex (NPC), which acts as a “molecular sieve” by selectively allowing the passage of specific proteins and other molecules [3]. The NPC is an impressive biological machine built upon a framework comprising multiple copies of roughly 30 different nuclear pore proteins called nucleoporins (Nups) [3,4]. The NPC represents one of the largest macromolecular complexes present in eukaryotic cells and spans the double lipid bilayer of the nuclear membrane, effectively creating an aqueous channel between the cytoplasm and nucleus. Nups within the NPC can be roughly categorized as either scaffold Nups, which function in an architectural capacity, or FG-Nups that make up the inner aqueous channel [4]. Selective interactions mediated by specific Nups provide the NPC with a diverse set of cellular roles, ranging from contributions to genome organization and architecture, gene expression, cytoskeletal tethering, and most notably, bidirectional transport of molecules across the NE [5,6,7].

Because of the size and composition of the NPC, molecules including proteins and RNAs cannot easily pass and therefore require assistance. The central channel of the NPC is a formidable gate-keeper, comprised of special FG-Nups, whose sequences are enriched with intrinsically disordered Phe and Gly repeats that form a hydrogel-like structure [8,9]. Lower molecular weight molecules, including small proteins, are capable of diffusing through the NPC so long as they can interact with FG-Nups. However, as protein size increases, the ability for a protein to passively diffuse through the NPC diminishes greatly [10,11]. Proteins that cannot simply diffuse into the nucleus employ the assistance of nuclear transport receptors, known as karyopherins, which serve as adaptors by facilitating interactions with FG-Nups [12,13].

## 2. The Nuclear Envelope Can Represent an Obstacle for Viral Infection

Understandably, the barriers that apply to cellular proteins and protein complexes also apply to intracellular pathogens, most notably viruses, whose nuclear functions are intertwined with cellular nucleocytoplasmic transport. For example, most DNA viruses and even some RNA viruses replicate their genomes within the host nucleus, and crossing the NE represents a barrier to infection and subsequent illness. As such, the mitotic state of the infected cell can become a key factor influencing the initial stages of infection, depending on the presence or absence of functional viral particle interactions with Nups [14]. As one example, a key difference between lentiviruses, like human immunodeficiency virus type 1 (HIV-1), and other types of retrovirus is the ability of the lentiviral preintegration complex (PIC) to cross the NE, allowing efficient infection of nonmitotic cells. A detailed review of mechanisms utilized by viruses to gain initial access to the nucleus was recently compiled by Kobiler et al. [15].

Once a virus that requires access to the nucleus begins its transcriptional program, export of virally encoded RNAs to the cytoplasm occurs, allowing translation of viral proteins by the host cell protein synthesis apparatus. Many viruses, whether nuclear or cytoplasmic, inhibit cellular mRNA nuclear export to prevent expression of cellular mRNAs encoding antiviral factors and to prevent host mRNAs from competing for access to the translation machinery. This preserves resources for viral protein production and often contributes to disease. Kuss et al. provide a detailed review of the diverse strategies used by viruses to alter or inhibit host cell mRNA export from the nucleus [16].

After translation, many newly synthesized viral proteins will traverse back through NPCs to the nucleus, and some will shuttle back and forth across the NE. These viral proteins may serve regulatory roles for viral and/or cellular gene expression, alter chromatin structure or integrity, participate in progeny virus assembly, and even trigger NE breakdown to allow viral progeny egress. In general, the appropriation of cellular nucleocytoplasmic transport machinery allows many viruses to replicate efficiently, can selectively alter host processes for the benefit of the virus, contribute to the evasion of the host antiviral immune response, and ultimately contribute to pathogenesis [17]. 

In this review, we discuss how different viruses can manipulate cellular components of the nucleocytoplasmic transport system in ways that allow them to control the transport of both viral and cellular molecules with an emphasis on protein trafficking into the nucleus. In addition, we will discuss how studies of viruses have provided, and continue to provide, mechanistic insight into nucleocytoplasmic transport of protein as well as opportunities to treat infection by targeting nuclear transport. 

## 3. Nucleocytoplasmic Transport of Cellular Proteins

**Figure 1:**
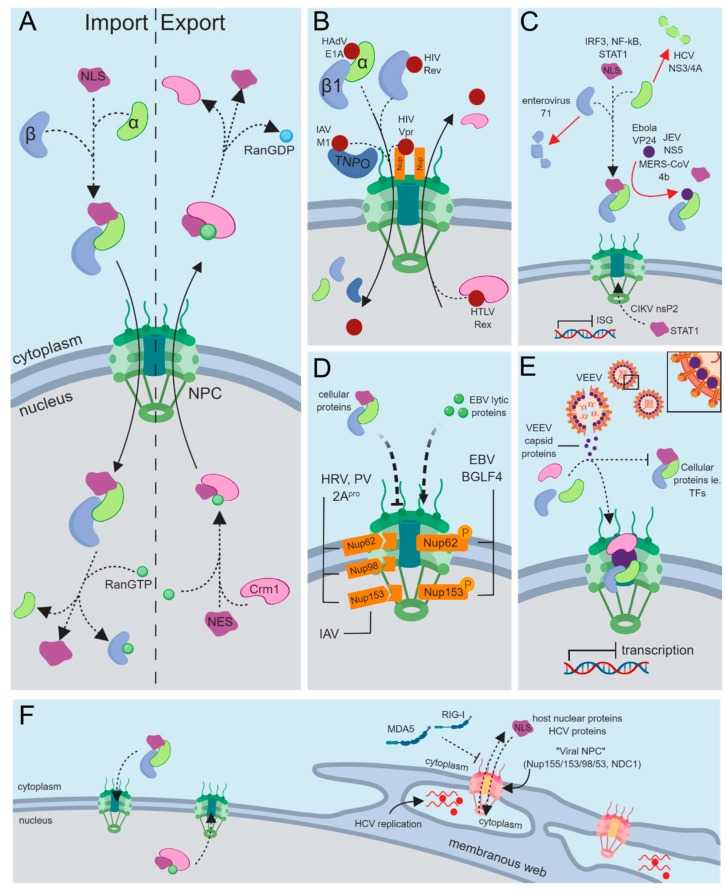
Viral appropriation of cellular nucleocytoplasmic transport. (**A**) Classical protein nuclear import mediated by Importin-α (Imp-α; green) and Importin-β (Imp-β; blue), and nuclear export pathways mediated by Crm1 (pink). (**B**–**F**) Selected examples of viruses perturbing or utilizing different components of the nuclear transport pathway. (**B**) Viruses can utilize the classical Imp-α/β pathway, Imp-β directly, the nuclear pore complex (NPC), or transportin through a PY-nuclear localization signal (PY-NLS) for nuclear import as well as Crm1 for nuclear export. (**C**) Viral proteins can perturb global nuclear transport by altering the dynamics of the NPC though the degradation or phosphorylation of nucleoporins (Nups). (**D**) Preventing nuclear import, or promoting export, of cellular proteins such as signal transducer and activator of transcription 1 (STAT1), interferon regulatory factor 3 (IRF3), or nuclear factor-kappa B (NF-κB) can block the antiviral innate immune response. (**E**) Venezuelan equine encephalitis virus (VEEV) capsid protein forms a tetrameric complex with Imp-α/β and Crm1 that “clogs” the NPC blocking import of other cellular proteins. (**F**) During hepatitis C virus (HCV) infection, key Nups are recruited to the membranous web, forming a “viral NPC”, to regulate transport of cellular and viral proteins as well as block access of pattern recognition receptors (PRRs) such as melanoma differentiation-associated protein 5 (MDA5) and retinoic acid-inducible gene I (RIG-I). Figure created with BioRender.

Passage of proteins through the NPC is often aided by transport factors called karyopherins, which can be functionally classified as either importins or exportins depending on the direction they carry their respective cargo. Most karyopherins belong to the highly conserved karyopherin-β (Kapβ) superfamily, which vary in number depending on the organism, ranging from 14 in the yeast *Saccharomyces cerevisiae* to at least 20 in humans [18]. Kapβ’s can bind their cargo directly through the recognition of either a distinct nuclear localization signal (NLS) or nuclear export signal (NES) [19]. Alternatively, they interact with cargo via adapter karyopherins, such as importin-α (Imp-α), which also recognize distinct NLS sequences [20]. Interactions between karyopherins and their cargo is dependent on a RanGDP/RanGTP gradient across the NE, with RanGTP being predominantly nuclear and RanGDP cytoplasmic [21,22]. Within the cytoplasm, importins are free to associate with their cargo. However, once in the nucleus, binding of RanGTP to karyopherins results in cargo dissociation. Exportins function in the reverse order, interacting with RanGTP and their cargo in the nucleus and dissociating from their cargo upon GTP hydrolysis within the cytoplasm (Figure 1A) [22]. 

To date, several mechanisms of protein nuclear import have been described, one of which is the classical nuclear import pathway. Classical nuclear import utilizes Imp-α as an adaptor for importin-β1 (Imp-β1) through binding of the Imp-β binding domain (IBB) within the N-terminal region of Imp-α [23]. This is the best characterized pathway and is assumed to handle the majority of protein nuclear import. In humans, seven different isoforms of Imp-α exist, each of which can bind a unique set of cargo. An advantage of using Imp-α as an adapter, despite this process being more energetically taxing, is an expansion of the repertoire of cargos that can indirectly utilize Imp-β1 [24]. The distribution of Imp-α isoforms across different cell types and at different stages of development is critical for normal cellular function [25]. Having this additional level of dynamic control would not be possible if proteins were only able to bind Imp-β1. Cargo proteins bind Imp-α through a classical NLS (cNLS), which is best exemplified by the viral Simian Virus 40 (SV40) Large T antigen (TAg) cNLS (PKKKRKV), one of the first NLSs ever described [26]. A common feature of all cNLSs is an abundance of basic amino acids and their relatively short sequence length. Additionally, these can be categorized as either monopartite, like the SV40 TAg cNLS, or bipartite, like with the nucleoplasmin cNLS (KRPAATKKAGQAKKKK) where two short stretches of basic amino acids (underlined) are separated by a linker region of varying length [27]. These sequence properties make cNLSs highly predictable, and this has led to the development of numerous computationally based NLS prediction algorithms [28]. 

Structurally, the interaction of cNLSs with Imp-α have been studied in detail [25]. Imp-α is composed of ten Armadillo (Arm) repeats that contain two NLS-binding grooves. The major NLS-binding groove is located within the N-terminal Arm repeats 2–4, and a minor site is located at Arm repeats 7–8. Monopartite cNLSs specifically bind to the major NLS-binding site of Imp-α, whereas bipartite cNLSs bind to both sites with the larger cluster of basic amino acids interacting with the major NLS-binding site [29,30]. 

Several other members of the karyopherin β family are involved in nuclear import, including the transportin proteins Imp-β2 (TNPO1) and Imp-β2b (TNPO2) as well as the bidirectional transporter importin-13 (Imp-13) [31]. Currently, only a limited number of Imp-βs have characterized NLSs for cargo binding. Of those identified, the best characterized NLSs are those that bind TNPO1 and TNPO2 using a PY-NLS. PY-NLSs can range from 15–100 residues long and are best described based on physical criteria, which include structural disorder, overall positive charge, an N-terminal hydrophobic/basic motif, and a C-terminal RX_2-5_PY consensus motif [32,33]. Interestingly, each of these characteristic motifs appear to be structurally independent and contribute uniquely to the binding interaction [34]. To date, many TNPO1 and TNPO2 cargos have been identified, most of which contain a PY-NLS [35]. Fascinatingly, some cargo proteins such as histone H3 are able to bind with high affinity without containing the PY motif and instead use an unusually strong N-terminal hydrophobic/basic motif [33]. 

Protein nuclear export mainly utilizes Crm1, otherwise known as Xpo1 or exportin-1, which recognizes leucine-rich NESs in the presence of RanGTP. These sequences are generally 8–15 amino acids long and contain 4–5 regularly spaced hydrophobic residues [36,37]. In fact, the first NESs identified were of viral origin, originally discovered within the Rev proteins of HIV-1 as well as other lentiviruses [38]. Hydrophobic residues within an NES act as anchors that bind a hydrophobic pocket formed by HEAT repeats 11 and 12 of Crm1 [39,40]. Unlike cNLSs, prediction of NESs is far less accurate, and this likely is due to a combination of several factors [39]. Specifically, NES binding to Crm1 is generally conformationally unrestrained because there is lack of contact with the NES backbone, which allows hydrophobic anchor residues to bind in a variety of conformations. In addition, NESs are unusual in that they are able to bind Crm1 in either the N- to C-terminal or C- to N-terminal orientation, further enhancing the diversity of potential NESs [39,40]. Export of mRNA, on the other hand, is typically handled by NXF1 via various adapter proteins, many of which are involved in mRNA processing, coupling processing with transport [41]. Several other members of the Imp-β family are associated with nuclear export of protein or RNA, and these include exportin-2, exportin-5, exportin-6, and exportin-7 as well as bidirectional transporters importin-13 and exportin-4. However, in contrast to Crm1, the cargo proteins recognized by these karyopherins have not been as extensively studied [31].

In summary, the diversity and range of nucleocytoplasmic transport processes provide the cell with multiple strategies for targeting cellular proteins to either compartment. Some of these pathways have been extensively studied, such as classical nuclear import and Crm1-mediate nuclear export, while others remain to be described in further detail. More recent examples of novel nuclear import pathways include those involving an ankyrin repeat:RanGDP pathway and transport of heat shock proteins using Hikeshi [42,43]. Together, these examples demonstrate the need for continued mechanistic investigation into nucleocytoplasmic transport, as novel strategies relating to transport and possible therapeutic intervention are likely to exist.

## 4. Targeting Nuclear Import Machinery through Viral Mimicry

Each component of the nuclear transport system potentially represents a viable target that can be appropriated by the virus during infection to allow and regulate entry of viral genomic information, export of viral mRNA, and passage of viral proteins bidirectionally across the NE. A common theme among many viruses is their limited coding capacity and, therefore, their absolute dependence on host proteins and pathways for a productive infection. Given this, it is unsurprising that viral proteins have evolved ways of interacting with the many components that make up the nuclear transport system.

### 4.1. Interactions with the Nuclear Pore Complex (NPC) 

The most direct approach for viral proteins to traverse the NE is to target the NPC itself. Generally, this phenomenon is reserved for capsid interactions to bring viral genomic information into the nucleus. Herpes simplex virus type 1 (HSV-1) UL36 is a preformed tegument protein, which aids in docking the viral capsid to the NPC by bridging the capsid with Nup358 of the NPC [44]. Similarly, the capsid protein of HIV-1 interacts with Nup153 to mediate import of the PIC [45]. Alternatively, human adenovirus (HAdV) has evolved an interesting mechanism for combining nuclear import of its genome with capsid disassembly. The hexon protein of the HAdV capsid interacts with Nup214, a filamentous protein of the NPC, which indirectly links to Kinesin-1 to cause disassembly of the viral capsid during passage through the NE [46]. For the most part, genomic studies have been primarily responsible for identifying components of the NPC important for the life cycle of many viruses; however, because of the nature of these experiments, it is often unclear which interactions directly involve the NPC [47].

Aside from viral capsids reaching the nucleus, many viral proteins themselves must be transported into and out of the nucleus by employing a variety of nucleoporins or karyopherins. Indeed, some viral proteins interact with the NPC directly, and several instances of viral proteins that are not components of the capsid directly binding the NPC have been documented (Figure 1B). These include BGLF4 from Epstein–Barr virus (EBV) and HIV-1 Vpr [48,49,50]. In vivo and in vitro experiments demonstrated that the C-terminus of BGLF4 can directly associate with Nup62 and Nup153 independently of other cellular factors. Based on structural predictions of BGLF4, its C-terminus may form multiple helical regions that allow it to interact with FG-rich Nups like Nup62 and Nup153. As Imp-β interacts with FG repeats through a series of HEAT repeats, which are also highly helical in nature, BGLF4 may mimic this structural property of Imp-β to cross the NE [51].

Vpr, on the other hand, interacts with a poorly characterized nucleoporin CG1, also known as nucleoporin-like protein 2 (NUPL2) [52]. During HIV-1 infection, Vpr is essential for nuclear import of the preintegration complex (PIC), an essential step for replication in nondividing cells that also involves Vpr binding Imp-α [53,54,55]. Docking the PIC at the NPC is facilitated by Vpr’s ability to bind the NPC through CG1 as well as Imp-α, essentially making it a functional mimic of Imp-β [56]. Binding the NPC is a strategy used by some cellular proteins, such as those involved in the Wnt signaling pathway like β-catenin, but other cellular examples of this transport mechanism are far less common compared to the number of cargos that use the classical transport receptors [57].

### 4.2. Interactions with Importins

Alternative approaches to using the NPC for nuclear import are exemplified by viral proteins that interact with karyopherins, specifically importins. These viral proteins make use of either Imp-α mediated import via the classic nuclear import pathway, surpass the adaptor in favor of a direct interaction with Imp-β1, or utilize other Imp-β family members to enter the nucleus. 

A simple yet effective approach to targeting cellular importins is through molecular mimicry of a cNLS, which would allow viral proteins to interact with Imp-α (Figure 1B). Because of the sequence characteristics and predictability of these peptide motifs, many viral cNLSs have been discovered in a diverse range of viruses. For example, influenza A virus (IAV) NP and PB2, HIV-1 integrase and Vpr, HAdV E1A, human papilloma virus (HPV) E2, HSV-1 pUL30, and many more all contain viral cNLSs [55,58,59,60,61,62,63,64]. Effectively, viral cNLSs fall into the category of protein–protein interaction modules mediated by short linear motifs (SLiMs) [65]. These motifs occur within intrinsically disordered regions of proteins and are typically 10 amino acids or less with only a few amino acids critical for binding, as is the case with only a few key basic amino acids within a cNLS. As a result of their low sequence complexity, changing a single amino acid can easily destroy or create a novel interaction through ex nihilo evolution [66]. These properties explain why SLiMs are ubiquitously found within cellular hub proteins such as p53, as well as viral hub proteins like HAdV E1A [65,67]. In general, molecular mimicry of SLiMs, which include cNLSs, is a common strategy used by viruses to perturb or usurp host cell networks [68,69]. These examples illustrate how viral proteins can mimic cNLSs at the amino acid level through convergent evolution. 

An alternative approach for viral proteins to target the nuclear import machinery is to directly bind Imp-β for entry into the nucleus (Figure 1B). Multiple viruses have been shown to bind Imp-β1 directly, surpassing the need for an adaptor protein. HIV-1 Rev was the first identified example of this, although similar examples can be seen with HIV-1 Tat, human T-cell leukemia virus (HTLV) Rex, HSV-1 capsid protein, hepatitis B virus (HBV) core protein, and HAdV VII [70,71,72,73,74,75,76]. Additionally, there are other members of the Imp-β family, namely transportins, which can mediate import of proteins into the nucleus. The IAV M1 and human cytomegalovirus (HCMV) UL79 proteins both contain PY-NLSs that allow their interaction with the transportin nuclear import pathway (Figure 1B) [77,78]. Alternatively, many viral proteins have been shown to utilize transportin; however, many have no identified PY-NLS. Specifically, HAdV core proteins V and VII, HIV-1 Rev, as well as HPV L2 and E6 have all been shown to interact with the transportin pathway, though none of these have identified PY-NLSs [73,76,79,80,81]. It remains unclear whether these proteins lack a PY-NLS or if they simply have an as yet unidentified PY-NLSs. 

While we have listed these mechanisms as separate examples, biological systems are rarely that simple. Both IAV and dengue virus (DENV) have been shown to have proteins that utilize multiple import mechanisms to enter the nucleus [82,83]. The DENV NS5 protein can make use of either classical nuclear import via Imp-α/β or through direct use of Imp-β for nuclear import. NS5 has a cNLS, which allows it to utilize the classical import pathway; however, NS5 is also capable of binding to Imp-β directly. Interestingly, this interaction can be prevented by another DENV protein, NS3 [82]. This competition may be related to the additional functions of NS5 in perinuclear viral genome replication, where the virus uses NS3 to reduce import of NS5, promoting its cytoplasmic accumulation [82]. IAV is another example of a virus using both the classical Imp-α/β pathway as well as direct use of Imp-β. The viral RNA polymerase is comprised of three subunits that make use of different importins for nuclear translocation. The A and B1 subunits dimerize within the cytoplasm before interacting with Imp-β3 for nuclear import, while the B2 subunit uses Imp-α3 for import [83]. Additionally, the B2 subunit can use different importins depending on its host (either avian or mammalian) for nuclear import. A unique domain in the B2 subunit, which is either folded or unfolded depending on the presence of a salt bridge, is responsible for this preference in importins [59,84]. These examples represent the complex interplay viruses have with host processes and how viruses will force the cell to permit nuclear access through one method or another. In addition to viral protein transport, viral interaction with importins also leaves room for potential negative regulation. In fact, Imp-α5 can bind to Imp-β1 in complex with the matrix protein of Newcastle disease virus, causing a reduction in overall nuclear import efficiency in the infected cells [85].

### 4.3. Nuclear Export and Nucleocytoplasmic Shuttling

As discussed above, many viruses gain access to the nucleus, for various reasons, via interactions with importins and the NPC. A consequence of having viral proteins and nucleic acids in the nucleus is that, unless the virus causes disassembly of the nuclear membrane, it needs to carefully manage the export of these components from the nucleus. Therefore, many viral proteins interact with Crm1 and the nuclear export machinery (Figure 1B). For example, HIV-1 Rev and HTLV-1 Rex, DENV NS5, HCMV pp65, respiratory syncytial virus (RSV) M protein, IAV NS2, and NP all utilize Crm1 to mediate nuclear export [86,87,88,89,90,91,92]. The main purpose of these proteins is to aid in nuclear export of viral mRNA, but there may be other potential reasons for manipulating export. 

Interestingly, some viral proteins contain both NLSs and NESs, and they are proficient at using both the import and export machinery. Some examples include HCMV UL94, HSV-1 VP19C, UL3 and US11, and HAdV E1A and E1B, which have at least one NLS and NES each [62,93,94,95,96,97,98,99]. An interesting phenomenon presents itself with viral proteins that are required to bidirectionally cross the NE. These proteins engage in so-called “nucleocytoplasmic shuttling”, which can require more complex regulation of transport signals. For example, IAV M protein and NP are capable of nucleocytoplasmic shuffling through their multiple NLS and NESs. While the M protein import is dependent on its NLS, its export is regulated by two N-terminal NESs as well as a C-terminal interaction with the viral NEP protein, all of which interact with Crm1 [100]. NP, on the other hand, makes use of at least one classical and one nonclassical NLS as well as two Crm1-independent NESs and one Crm1-dependent NES [100]. An even more complex example can be seen with rabies virus, which uses multiple isoforms of its P protein to regulate nuclear import and export. P protein has a C-terminal NLS as well as an N-terminal NES, which overlaps an additional NLS. In isoforms that are N-terminally truncated, the NLS overcomes the NES function leading to a largely nuclear accumulation of the protein [101]. In general, this shuttling phenomenon is likely important to the completion of each respective viral lifecycle. West Nile Virus (WNV) NS5 is a definitive example of the importance of nucleocytoplasmic shuttling for productive infection, as mutation of its NES impairs viral replication [102]. Taken together, numerous examples of viral proteins that contain both an NLS and NES exemplify the importance of regulating bidirectional transport across the nuclear membrane during infection.

## 5. Viral Disruption of Cellular Protein Trafficking

For efficient replication, a virus’s replicative cycle requires cellular proteins to be correctly localized within the cell. RNA viruses that replicate strictly in the cytoplasm, like picornavirus, often require host proteins that normally reside in the nucleus. Likewise, DNA viruses that replicate in the nucleus, like HAdV, can relocalize normally cytoplasmic proteins. Spatial reorganization of the proteomic landscape of a cell may allow viruses to evade cellular antiviral processes, including innate immunity, as well as collect the appropriate resources into the correct subcellular compartment necessary for the viral replicative cycle.

### 5.1. Subverting Antiviral Immunity through Nucleocytoplasmic Transport

The host’s innate immune response serves as a front-line defense against viral infection [103]. This relies on the recognition of viral components by cellular proteins called pattern recognition receptors (PRRs), which trigger a signal transduction cascade that causes the infected cell to mount an innate immune response [103]. Central to the innate antiviral immune response is the activation of type I interferon (IFN) and NF-κB signaling pathways, which lead to the expression of IFN stimulated genes (ISGs) and the production of an array of proinflammatory cytokines that ultimately work to halt or delay viral replication. The production of type I IFN is triggered by the relocalization of interferon regulatory factor (IRF) 3, IRF-7, and/or NF-κB transcription factors from the cytoplasm to the nucleus in response to activated PRRs. The canonical type I IFN response involves activation of the Janus kinase (JAK) and signal transducer and activator of transcription (STAT) pathways upon binding of IFN to its receptor. This triggers translocation of activated STAT proteins into the nucleus resulting in transcription of hundreds of ISGs [104]. In addition to contributing to type I IFN induction, the NF-κB signaling pathway induces expression of a wide variety of other proinflammatory innate immunity responses [105]. NF-κB is composed of a family of transcription factors (p50, p52, p65, c-Rel, and RelB). Classical activation of this pathway involves the release of p50/p65 from an inhibitory complex with the inhibitory component IκBα in the cytoplasm, allowing p50/p65 to translocate into the nucleus and activate expression of a variety of proinflammatory genes [106]. The evolutionary relationship between viruses and their host has led viruses to develop strategies that allow them to subvert host antiviral immune responses. Since many of the signaling proteins involved in the IFN response require passage across the NE, it is not surprising that viruses have evolved strategies to target the different components involved in nucleocytoplasmic transport as a means of blocking these antiviral processes (Figure 1C).

One component that can be targeted to block innate antiviral responses is the karyopherin proteins, which are directly responsible for cargo recognition. An example of this is the hepatitis C virus (HCV) NS3/4A protease, a well-studied protein involved in evading innate immunity [107]. NS3/4A functions by interacting with and subsequently cleaving Imp-β1, resulting in its loss of function [108]. This study also demonstrated that Imp-β1 was the main nuclear import receptor for interferon regulatory factor 3 (IRF3) and NF-κB, which are key transcription factors controlling antiviral innate immunity as described above. Thus, cleavage of Imp-β1 reduces nuclear transport of IRF3 and NF-κB p65 at early time points during infection, effectively delaying the IFN response. As another example, Imp-α is degraded in a caspase-3-dependent manner during enterovirus 71 infection, preventing NF-κB function [109,110]. While many other viruses have evolved strategies to suppress NF-κB activity, cleavage of Imp-α or Imp-β1 by a viral protein represents a novel strategy to block NF-κB from performing its nuclear functions by interfering with its transport across the NE. 

A less direct way of blocking nuclear translocation of cellular proteins involved in innate immune signaling is through simple competition for Imp-α. Ebola VP24 can compete with tyrosine-phosphorylated STAT1, whose nuclear import is essential for transcriptional activation of ISGs, for binding to Imp-α5, α6, and α7 [111,112]. Japanese encephalitis virus NS5 protein is able to bind Imp-α3 and α4 to compete with IRF3 and NF-κB p65 binding, preventing their nuclear import and ability to activate IFN signaling [113]. More recently it was shown that the 4b protein of Middle East respiratory syndrome coronavirus is able to out compete NF-κB p65 for binding to Imp-α3 [114]. Beyond the examples listed here, other viruses have evolved similar mechanisms to target importins to evade host innate immunity. This can include sequestering importins in the cytoplasm and even expressing microRNA to downregulate Imp-α expression [17,115,116]. As mentioned previously, how viruses can alter the mRNA export pathway to promote viral mRNA and/or block host mRNA export has been reviewed [16]. However, examples of viruses targeting the protein nuclear export pathway mediated by Crm1 to specifically evade antiviral immunity are less common. As one example, the methyltransferase-like domain of Chikungunya virus (CHIKV) nsP2 protein promotes nuclear export of STAT1 through a currently unknown mechanism. Taken together, these examples demonstrate that targeting karyopherins (importins/exportins) can be a key step in immune evasion and presumably viral pathogenesis. 

A completely different approach used by a variety of viruses to evade host innate immunity is the establishment of viral organelle-like replication factories (RFs) [117,118]. RFs are specialized membranous compartments that form in the nucleus or cytoplasm, depending on the virus, where necessary cellular factors are recruited and concentrated for efficient virus replication. Additionally, these membranous structures shield the viral genome from the hostile cellular environment and/or detection by PRRs. In the case of HCV, which forms a type of RF referred to as cytoplasmic membranous webs (MWs), essential Nups and karyopherins are recruited to these structures to form a derivative of a functional NPC (Figure 1F) [119]. Moreover, during HCV infection, GFP-SV40 TAg cNLS fusions can specifically localize to MWs, demonstrating functional NPC-mediated transport. Upon infection, expression of a subset of Nups was also increased, suggesting that these cytoplasmic NPCs are being built from newly synthesized Nups as well as appropriating them from existing Nup pools. The use of these NPC derived structures to regulate transport allows cellular proteins with NLSs to shuttle into the MW, while excluding cellular antiviral sensors like retinoic acid-inducible gene I (RIG-I) and melanoma differentiation-associated protein 5 (MDA5), both which recognize viral RNA and do not have NLSs [120,121]. Under normal cellular conditions, cytoplasmic functions for karyopherins and NPCs have been observed with annulate lamellae, a structure composed of stacked endoplasmic reticulum-derived membranes [122,123]. These structures have also been observed to accumulate during infection with viruses like hepatitis A virus, Japanese encephalitis virus, and rubella virus. However, the role of annulate lamellae during viral infection has largely remained elusive [123,124,125,126]. 

### 5.2. General Disruption of Host Nucleocytoplasmic Transport

Beyond evading innate immunity, the viral replicative cycle may require localization of cellular proteins to subcellular compartments different from their normal localization. In some cases, relocalization is specific to certain proteins, while in others it is broad effecting a multitude of proteins. Picornaviruses, such as human rhinovirus (HRV) and poliovirus (PV), are able to target Nup153, a component of the NPC nuclear basket, as well as the FG-rich nucleoporins Nup62 and Nup98 causing their cleavage through virally encoded 2A proteases (2A^pro^) (Figure 1D) [127,128,129,130]. More specifically, 2A^pro^ from HRV and PV specifically cleaves the FG-rich region from Nup62, a region involved in recognizing karyopherins and forming the inner channel of the NPC [131]. Applying quantitative mass spectrometry to HRV-infected cells, roughly 276 proteins with increased abundance in the cytoplasm were identified. This appeared to be a highly selective re-equilibration of proteins, with most of the affected targets having known mRNA splicing and transport-related functions. Importantly, at least some of these normally nuclear factors contribute to virus replication in the cytoplasm, suggesting that these viruses systematically hijack these functions via interference with the NPC, leading to subcellular relocalization of key cellular factors that promote infection [132]. 

Although less well-studied, other viruses such as IAV, EBV, and Venezuelan equine encephalitis virus (VEEV) have evolved strategies for targeting components of the NPC as well. EBV, a DNA virus, encodes the Ser/Thr protein kinase BGLF4, which induces phosphorylation of Nup62 and Nup153 (Figure 1D) [133]. In the presence of BGLF4, nuclear targeting of Imp-β1 was attenuated, inhibiting cNLS-mediated nuclear import in general. In contrast, the nuclear import of several non-NLS containing EBV lytic proteins was promoted in the presence of BGLF4. IAV, an RNA virus that replicates within the nucleus, induces enlargement of the NPC by exploiting cellular caspase activity [134]. Caspase activation at later timepoints during infection results in degradation of Nup153, altering the structural integrity of the NPC and likely aiding in passive diffusion of ribonucleoprotein complexes through the nuclear envelope. Another RNA virus with an unusual strategy of targeting the NPC is VEEV, a mosquito-borne pathogen that can affect humans in addition to equine species. Amino acid region 30–68 (H68) of the VEEV capsid protein contains both an NLS and NES, which allows it to simultaneously bind the opposing transport factors Crm1 and Imp-α/β [135]. The NES, in particular, is unique in that it strongly binds Crm1 within the cytoplasm in the absence of RanGTP. In an attempt to shuttle into the nucleus, this tetrameric complex becomes wedged within the NPC, essentially “clogging” it (Figure 1E). Both the H68 peptide and capsid protein appear to have the same effect on classical nuclear import by blocking transport of cNLS-bearing recombinant proteins in addition to transcriptional shutoff [135,136]. How exactly this is achieved, which Nups are targeted and the cellular effects of blocking NPCs during infection, remains to be further characterized. 

Establishing a state conducive to viral infection can also involve precise nucleocytoplasmic redistribution of select cellular proteins, a process best described by the HAdV E1A protein. E1A itself has no intrinsic DNA binding or enzymatic capabilities and therefore relies on host proteins to carry out such functions [67]. During HAdV infection, E1A interacts with the regulatory subunits RIα and RIIα of protein kinase A (PKA) and preferentially relocalizes them to the nucleus from the cytoplasm. Interestingly, HAdV-5 and -12 have different preferences, with the former relocalizing RIα and the latter RIIα [137,138,139]. Normally, the subcellular localization of PKA is regulated by cellular A-kinase anchoring proteins (AKAP). However, during infection, E1A appropriates this role by direct competition with cellular AKAPs and functions as a viral AKAP (vAKAP) mimic [137,139]. PKA is a major Ser/Thr kinase with hundreds of documented phosphorylation sites listed in PhosphoSitePlus, and by altering the subcellular localization of PKA the phosphorylation status of many cellular proteins is likely effected [140]. 

Collectively, these examples demonstrate how viruses can target the different components of the nuclear transport system to cause widespread or selective spatial redistribution of cellar proteins. Targeting the NPC is a common strategy adopted by diverse viruses to alter host nucleocytoplasmic transport or even establish its own transport system, like with HCV, while other viruses like HAdV can relocalize single proteins to the nucleus. By disrupting transport between the cytoplasm and nucleus, viruses can elicit large proteomic changes that influence cellular dynamics in ways that are beneficial to the viral life cycle and promote infection.

## 6. Viruses as a Tool to Discover Mechanisms of Nuclear Transport

Classical nuclear import has been studied extensively and is assumed to handle the majority of protein import into the nucleus [141]. This is likely due to the number of proteins with documented cNLSs, as well as the sequence characteristics of cNLSs, which make them highly amenable to computational prediction [142,143,144,145]. Despite this, prediction programs fail to identify cNLSs in 40–60% of nuclear proteins from both yeast and mice. In addition, up to 50% of proteins that interact with Imp-α in yeast do not have a predictable cNLS [28,141]. Taken together, these findings highlight the fact that many nuclear proteins in yeast, mice, and likely humans, are transported to the nucleus using nonclassical pathways, unusual NLSs, or possibly by using the classical pathway in a nonclassical or unconventional manner. 

One limitation when studying nonclassical nuclear import is the lack of definitive consensus motifs compared to cNLSs. This makes identifying potential NLSs in both cellular and viral proteins difficult, even when they are known to localize to the nucleus. Characterizing the nuclear import of viral proteins can provide useful insight into novel nuclear import strategies. One such example of this was revealed by the structure of Imp-α bound to IAV NP [146]. NP binds exclusively to the minor NLS-binding site in Imp-α, a site typically occupied by bipartite cNLSs. Why NP specifically targets the minor site and the consequences of this specific mode of binding to Imp-α remains to be determined. It is plausible that by binding the minor NLS-binding site, NP can compete with cellular bipartite cNLSs for Imp-α without influencing the binding of monopartite cNLSs to the major NLS-binding site. Binding the minor site occurs within the extreme N-terminal region of NP, with the amino acid sequence SQGTKRSYEQME. This sequence was originally described nearly two decades ago as one of the first nonclassical NLSs and is not identified by common cNLS prediction algorithms [147]. Other cellular proteins since then have been shown to preferentially bind Imp-α’s minor NLS-binding site; however, the unconventional NP NLS only weakly resembles those cellular sequences [142,148,149]. Defining rules that can identify peptide sequences that bind the minor NLS-binding site is currently under way [150]. Thus, through the study of viral proteins such as IAV NP, these rules can be further refined while also providing mechanistic knowledge as to the consequences of these binding preferences.

Like IAV NP, several other viral NLSs do not adhere to the rules defining a cNLS. An interesting example of this is the HSV-1 VP19C NLS (PRGSGPRRAAS) [97,151]. Nuclear import of VP19C depends on Imp-β1 but does not require Imp-α. It is unknown if VP19C directly interacts with Imp-β1; however, it also remains possible this NLS functions by piggybacking on other Imp-β1 binding proteins. As another example, the Borna disease virus (BDV) p10 protein NLS (RLTLLELVRRLNGN) can bind Imp-α1 both in vitro and in vivo [151]. This NLS, like the VP19C NLS, harbors a small cluster of basic amino acids that are characteristic of a cNLS yet is not sufficient to define it as a cNLS by conventional criteria. Additionally, the abundance of leucine in this motif is intriguing, as it makes this nonclassical NLS more characteristic of a nuclear export signal. Additionally, this same stretch of amino acids binds to the virally encoded BDV p24 phosphoprotein, setting up an equilibrium that may regulate localization or function [152]. The unique characteristics of this motif demonstrate a dual-purpose approach to achieving nuclear import and forming other protein–protein interactions. 

In addition to what viral NLSs teach us about nonclassical or unconventional nuclear import, some viral proteins have expanded our knowledge of already well-established cNLSs. Based on the nucleoplasmin bipartite cNLS, the linker region between the two clusters of basic amino acids defining a bipartite cNLS have been traditionally limited to a maximum of ten amino acids [27]. Studies of the Ty1 integrase protein, an essential component of the yeast *Saccharomyces cerevisiae* Ty1 retrotransposon (considered a primitive retrovirus), showed that it contained a bipartite cNLS with a 29 amino acid linker [153,154,155]. Having evidence of a functional bipartite cNLS, albeit of viral origin, with an extended linker region led Lange et al. to search the yeast proteome for similar cellular bipartite cNLSs. This allowed them to experimentally confirm that Rrp4 had a true bipartite cNLS with a linker of 25 amino acids, demonstrating how studies of viral proteins can provide insight into cellular protein nuclear import.

As linker length increases, so does the probability of finding new bipartite cNLSs, or in some instances redefining a previously defined monopartite cNLSs as bipartite. This was the case with the HAdV-5 E1A protein, whose C-terminal cNLS was historically described as monopartite (KRPRP) [61,156]. Upon further examination of the E1A C-terminal region, the monopartite cNLS was predicted and experimentally determined to be a bipartite cNLS with a 21 amino acid linker region [62]. Interestingly, within this bipartite cNLS linker region lies a CtBP binding motif (PLDLS) [157]. Finding other protein interaction motifs within the linker region between the two halves of a bipartite cNLS is an uncommon feature. To our knowledge, the only other example of this is the *S. cerevisiae* Whi5 protein, which harbors a binding motif for the proline isomerase Ess1 in the bipartite NLS linker [158]. It remains unknown whether or not these binding sites compete or assist with Imp-α interaction. 

In contrast to already established cNLSs, there are regions within viral proteins that have no resemblance to a prototypical cNLSs. The HCMV pUL34 protein is one such example of this, with a peculiar NLS (QTPHMWARSIRLI) spanning amino acids 198–210 [159]. Interestingly, cNLS prediction software predicted two possible NLSs in this protein, neither of which were present in the 198–210 fragment. Using pUL34-GFP fusion proteins, deletion of this region resulted in loss of nuclear localization, even when the other predicted cNLSs were present. Other examples like pUL34 exist within the HAdV E1A proteins across multiple different species [160]. Using a yeast-based nuclear import assay, amino acids 30–69 of E1A were shown to mediate nuclear import. This region indirectly interacts with two different representative Imp-α family members Qip1 (Imp-α2 family member) and Rch1 (Impα-1 family member), despite lacking any basic residues. These results suggest that a piggybacking mechanism allows E1A to localize to the nucleus via a mechanism separate from the bipartite cNLS described above. As mentioned before, many nuclear proteins in yeast that bind Imp-α do not have a predictable cNLS, making piggybacking into the nucleus a likely strategy for cellular proteins as well [28,141]. The extent to which piggybacking into the nucleus is utilized as the sole method of nuclear import remains unknown, and there are only a few confirmed examples of cellular proteins utilizing this strategy [161,162,163,164]. However, the evidence provided by viral proteins such as E1A suggests that this could be a more common process employed by cellular proteins than previously thought [165].

Much like the SV40 TAg NLS, one of the first NLSs ever described, viral nuclear import strategies can provide researchers with tools to understand mechanism and find parallel cellular processes. Many of the molecular mechanisms underlying these unusual or peculiar NLSs have yet to be further explored, and it is likely other viral and cellular examples like these exist. With the aid of viral proteins, our definition of cNLSs (and NLSs in general) is expanding, shedding light on novel cellular nuclear import strategies.

## 7. Targeting Nuclear Transport to Control Viral Replication

Despite substantial differences in the viral replicative cycle between RNA and DNA viruses, both classes of viruses utilize components of the nuclear transport system. Indeed, nuclear import and export of viral proteins and RNA is required for efficient replication of many viruses. Regardless of how different viruses utilize the nuclear transport machinery, the reliance of many viruses on nuclear import and export make it a logical therapeutic target for treating infection. To date, several drugs have been shown to target either nuclear import or export, many of which are in clinical trials and some of which are already approved for certain indications unrelated to viral infection [166]. 

One of the best documented drugs impacting the nuclear import process is ivermectin (IVM). Currently, IVM is one of the most widely distributed antiparasitic drugs and has contributed to a dramatic decrease in river blindness and lymphatic filariasis in some of the poorest parts of the world [167]. As a drug already approved for human use, with minimal side effects, IVM has become of increasing interest as it represents an attractive prospect for repurposing for treatment of other illnesses. Indeed, IVM may have therapeutic benefits in other diseases, including nonalcoholic fatty liver disease, inhibition of cancer stem-like cells, and antitumor effects through targeting several pathways involved in cancer [168,169,170]. More recently, IVM was shown to reduce the incidence of childhood malaria as well as provide protection through its mosquitocidal effects [171,172]. In regards to nucleocytoplasmic transport, a high-throughput drug screen specifically targeted at identifying inhibitors of nuclear import yielded IVM as a broad-spectrum Imp-α/β inhibitor [173]. 

With respect to antiviral function, IVM is effective against RNA viruses, including HIV, as well as mosquito-borne flaviviruses like DENV and alphaviruses like CHIKV [174,175,176]. Studies of the antiviral function of IVM have shown that it is a potent nuclear import inhibitor of HIV-1 integrase as well as DENV-2 NS5 protein, and that treatment with IVM inhibits infection with HIV-1 and DENV-2 [176]. Additionally, IVM was shown to be an inhibitor of NS5 nuclear import, and it protected against infection from all four DENV serotypes [174]. Currently, dengue fever is considered one of the most common and rapidly spread mosquito-borne viral diseases and is endemic to more than 100 countries [177]. The incidence of DENV infection over the past 50 years has increased 30-fold, demonstrating the need for prevention and control strategies [178,179]. DENV is primarily spread by *Aedes* mosquitos, which include *A. aegypti* and *A. albopictus*. It was also recently demonstrated that treatment of infected *A. albopictus* mosquitos with IVM inhibited viral replication of DENV-2, suggesting IVM could be a valuable strategy for controlling mosquito-borne infection and spread of DENV [180]. 

IVM has also shown potency against other flaviviruses like Zika virus (ZIKV), which currently has no approved vaccines or specific treatments [181]. A screen of FDA-approved drugs demonstrated IVM to be an effective inhibitor of ZIKV infection across multiple cell types with minimal toxicity [181]. Furthermore, these studies demonstrate that IVM can be effective against a genus of virus, like flavivirus, and potentially others, such as alphavirus, where IVM treatment of infected cells resulted in a 2–4 log reduction in viral titers from CHIKV, Semliki Forest virus, and Sindbis virus [175].

Beyond IVM, other nuclear import inhibitors have not been studied as extensively for targeting the viral life cycle. The steroid analog mifepristone (marketed as Mifegyne), another importin α/β inhibitor, has been shown to inhibit adenovirus infection as well as the nuclear import of HIV-1 integrase and the VEEV capsid protein [182]. Based on this mounting evidence, Imp-α/β inhibitors may have useful clinical activities for targeting viral infections [173,183]. Currently, neither IVM nor mifepristone is approved for treatment of any viral-based infections despite their potential therapeutic benefit. In another example, high-throughput drug screening discovered several compounds that were able to target the VEEV capsid protein:Imp-α/β interaction [184]. Unique to these compounds were their abilities to specifically inhibit this interaction without blocking the TAg-cNLS:Imp-α/β interaction, essentially leaving classical nuclear import unscathed. 

In contrast to nuclear import, the development of inhibitors targeting protein nuclear export via Crm1 is far more advanced. The first drug discovered to inhibit nuclear export was the antibiotic leptomycin B (LMB), which was originally shown to inhibit nuclear export of the HIV-1 Rev protein [185]. Despite LMB administration being highly toxic in humans, its use a molecular tool has aided in the discovery of hundreds of Crm1 export cargos and continues to be a valuable tool for studying nucleocytoplasmic shuttling [186,187]. Many drugs have been shown to target Crm1-mediated export; however, none have been tested in a clinical setting to treat viral infection [188]. A more recent class of drugs under development are the selective inhibitors of nuclear export (SINE), which are primarily being investigated as anticancer agents and are involved in several clinical trials [189]. Of the several drugs in this class, Verdinexor (KPT-335) has been demonstrated to function as a potential antiviral [190]. In vitro evidence has shown the effectiveness of Verdinexor against RSV, VEEV, IAV, EBV, Kaposi’s sarcoma virus (KSHV), HAdV-5, and HPV-11 [191,192,193,194]. Additionally, prophylactic and therapeutic administration of Verdinexor protected mice against disease pathology when challenged with IAV [193]. Similarly, Verdinexor-treated ferrets had reduced viral burden and lung pathology associated with IAV infection [195].

Examples such as those described above highlight nucleocytoplasmic transport as a potential avenue for treating viral infection and, more importantly, doing so with minimal toxicity. Based on our current knowledge, targeting protein nuclear import or export by repurposing drugs like IVM and Verdinexor could be a viable approach for treating and controlling viral infections.

## 8. Conclusions

As obligate intracellular parasites, viruses are critically dependent on numerous host cell proteins and pathways for their replicative cycle. Many viruses rely on the host nucleocytoplasmic transport apparatus, taking advantage of both classical and nonclassical mechanisms to gain access to and from the nucleus. Through targeting certain components of the nucleocyctoplasmic transport machinery, viruses can have profound effects on the cellular proteome to manipulate cellular processes including innate immunity. In general, studying the relationships of viruses and their hosts continues to teach us about underappreciated aspects of nucleocytoplasmic transport and may provide new targets for the control of virus infection and their associated diseases.
